# Action and familiarity effects on self and other expert musicians’ Laban effort-shape analyses of expressive bodily behaviors in instrumental music performance: a case study approach

**DOI:** 10.3389/fpsyg.2014.01201

**Published:** 2014-10-29

**Authors:** Mary C. Broughton, Jane W. Davidson

**Affiliations:** ^1^School of Music, The University of QueenslandBrisbane, QLD, Australia; ^2^Melbourne Conservatorium of Music, The University of MelbourneMelbourne, VIC, Australia

**Keywords:** music performance, bodily action, embodied cognition, Laban effort-shape analysis, expression

## Abstract

Self-reflective performance review and expert evaluation are features of Western music performance practice. While music is usually the focus, visual information provided by performing musicians’ expressive bodily behaviors communicates expressiveness to musically trained and untrained observers. Yet, within a seemingly homogenous group, such as one of musically trained individuals, diversity of experience exists. Individual differences potentially affect perception of the subtleties of expressive performance, and performers’ effective communication of their expressive intentions. This study aimed to compare self- and other expert musicians’ perception of expressive bodily behaviors observed in marimba performance. We hypothesized that analyses of expressive bodily behaviors differ between expert musicians according to their specialist motor expertise and familiarity with the music. Two professional percussionists and experienced marimba players, and one professional classical singer took part in the study. Participants independently conducted Laban effort-shape analysis – proposing that intentions manifest in bodily activity are understood through shared embodied processes – of a marimbists’ expressive bodily behaviors in an audio-visual performance recording. For one percussionist, this was a self-reflective analysis. The work was unfamiliar to the other percussionist and singer. Perception of the performer’s expressive bodily behaviors appeared to differ according to participants’ individual instrumental or vocal motor expertise, and familiarity with the music. Furthermore, individual type of motor experience appeared to direct participants’ attention in approaching the analyses. Findings support forward and inverse perception–action models, and embodied cognitive theory. Implications offer scientific rigor and artistic interest for how performance practitioners can reflectively analyze performance to improve expressive communication.

## INTRODUCTION

The current paper contributes new knowledge to the field of expressive body movement research in music. Specifically, it demonstrates that observers’ specific motor experience may affect the degree of information available to them in appraising music performances. Why is this research important? Performing musicians engage in processes of self-reflection and performance review in the cycle of continuing development. Self-reflection is recognized as a key phase of self-regulated learning, following phases of forethought, and performance (Zimmerman, 2002). Self-reflective performance practice foci include refining technical skills and improving communication of musical interpretation and expression. However, Bergee (1997) observed that undergraduate music student self-evaluation of performance, using categories drawn from an established assessment scheme in the USA, correlated poorly with faculty and peer evaluations. The self-evaluations did not significantly differ between different instrumental areas of study (e.g., wind, strings players, etc.), or between lower and upper years of undergraduate study programs. However, inter-judge scores revealed divergent assessments.

Similarly, Thompson and Williamon (2003) observed differences in expert musicians’ evaluations of performance, using an established assessment scheme in the UK, according to whether or not the expert musician was evaluating performance from their own instrumental group (e.g., strings). These lines of research raise important issues for performance practice and training. Firstly, music performance assessment schemes may not adequately account for factors that are potentially influential in performance quality judgments, such as expressive movement behaviors. Secondly, the possibility that individual differences in embodied experiences, such as instrument-specific motor expertise, account for differences between self and other, or between expert panel members’ performance evaluations. Together these lines of enquiry require thorough investigation.

The current paper makes a novel contribution to this field, examining the nexus between self and others’ performance evaluations, through the lens of embodied cognition, focussing on analyses of a performing musician’s expressive bodily behaviors. The present study makes use of the recorded performance of an expert marimba player in the Western classical tradition. Marimba performance was selected to study as playing the marimba involves a relatively high degree of physical and spatial movement in comparison to other instrument types, such as the flute. Furthermore, the movements necessary to play the marimba are obvious, occurring external to the body, and thus closely allied to the produced sounds. Self-analysis, using the effort-shape analytical system (Broughton and Stevens, 2012), is compared to analyses conducted by two other expert musicians – one a percussionist, the other a singer. We expect that individual differences in embodied expertise to help explain differences between participants’ effort-shape analyses. The focus of the present study is on expressive bodily behaviors, as these have been shown to affect a range of observers’ judgments of music performance.

This paper also makes a new contribution to a growing corpus of research taking a practice-led approach to investigating music performance. Haseman (2006) proposes *performative research* as a third research paradigm, contrasting with traditional quantitative or qualitative paradigms and methodologies, which centralize practice within the research process. Performative research can include participant, participatory, collaborative, and action research strategies, and utilize reflective, observational, expert and peer review methods, and personal experience. Complementing research findings expressed as quantitative and qualitative symbolic data are other symbolic data presented as or through the medium of practice, working to vivify performed actions and sometimes stand for the research itself. For example, these other symbolic data might be forms of images, music, live action, or digital code. In the field of music performance research, the value of the practice-led or performative research approach is being recognized in traditional modes of academic publication.

Journal and book chapter publications have demonstrated how musicians can make valuable contributions to music performance research investigations in dual roles: (i) as performer and the subject of investigation, and (ii) as researcher and contributing author (e.g., creative collaboration – Barrett et al., in press; expert memory – Chaffin and Imreh, 2002; Ginsborg et al., 2006; Ginsborg and Chaffin, 2011). In collaborative investigations of this nature, the insights provided by the performing musician-researcher(s) are checked and balanced by a co-author, who brings a more objective perspective to the investigation. The present study sits within this practice-led performative research paradigm. The first author’s contribution as reflective practitioner and researcher is complemented and balanced by the second author’s less subjective involvement.

In the following section we reference studies of instrumental musicians, as these are most relevant to the present investigation. In contrast to singers, instrumental musicians have an object they physically manipulate to produce sound. Therefore, they are relatively less free to use their bodies expressively than singers who are less encumbered in this manner. Furthermore, as sung music often contains lyrics, language potentially affords singers another stream of expressive communication.

### MUSICIANS’ EXPRESSIVE BODILY BEHAVIORS AFFECT JUDGMENTS OF PERFORMANCE

Experiment-based research demonstrates that instrumental musicians’ expressive bodily behaviors have an effect on observers’ judgments of performance. For example, Dahl and Friberg (2007) showed that observers could detect solo musicians’ emotional expressive intentions from vision-alone displays of them performing with happiness, sadness, and anger, although not fear. In multi-modal experiments, the visual information provided by solo musicians’ performing bodies has been shown to influence observer’s perception of auditory information, affecting judgments of note duration (Schutz and Lipscomb, 2007), expressiveness (Davidson, 1993), tension and phrasing (indicators of emotion and structural segmentation, Vines et al., 2006), as well as judgments of music elements such as rubato and dynamics (Juchniewicz, 2008). As well as affecting expressive judgments of performance, Broughton and Stevens (2009a) found that observer interest in solo marimba performance was higher when they could see, as well as hear, the musician performing in a projected, public performance manner.

With research demonstrating the significant effect of musicians’ bodily behaviors on a range of performance-related judgments, it is crucial for musicians to understand how observers might perceive their performance through visual as well as auditory senses. However, while musicians might engage in self-reflective performance development practices and review recordings of their performances, their attention is typically focused on the music, rather than on allied bodily behaviors. Furthermore, even if some consideration is paid to the effects their expressive bodily behaviors might have on observers, musicians might be less likely to consider how observers with different experiences might respond differently to their embodied expressive intentions. While musicians’ expressive bodily behaviors can affect observers’ judgments of performance in general ways, regardless of music training (e.g., judgments of note duration, Schutz and Kubovy, 2009; or tension and phrasing, Vines et al., 2006), in other situations observers’ music training can shape their responses to performance presentations.

### EXPERTISE-MODERATED JUDGMENTS OF MULTI-MODAL MUSIC PERFORMANCE PRESENTATIONS

Experiment-based research has demonstrated that in addition to the performing musicians’ expressive bodily behaviors, observers’ music expertise affects how they judge performance. For example, Broughton and Stevens (2009a) found that musically trained participants rated excerpts of solo marimba performance higher on expressiveness and interest dependent variables than musically untrained participants. In another multi-modal study in which stage behavior was manipulated (minimum, natural, and exaggerated), Huang and Krumhansl (2011) observed that musically trained participants were able to detect the pianist’s different levels of stage behavior in audio-only and audio-visual conditions. However, musically untrained participants were only able to detect difference in stage behavior in the audio-visual condition. Furthermore, the style of the music appeared to elicit preferences for a certain style of stage behavior. Overall, non-musicians’ summary and structural ratings were lower than their emotional ratings, or all those given by the musicians. These studies demonstrate that additional to what and how a musician might perform, the observer brings to the task of judging performance their own music expertise, which exerts an influence on their judgments.

In studying the effects of variables on measures of performance, performance quality might be only a component, or an implied attribute, of investigations. From a music performance perspective, assessing performance quality is a normal practice in the artistic domain. Several studies have considered factors that may influence judgments of performance quality, such as observers’ music expertise.

Investigations of music performance quality assessment indicate that musically trained observers’ own specialist music expertise may also affect their judgments of performance. Wapnick et al. (2004) conducted an experiment-based study in which musically trained observers judged excerpts of solo piano performance recorded at an international piano competition. Observers rated performance excerpts in either an audio-only or audio-visual condition along six dimensions commonly used in music performance assessment. Overall, audio-visual excerpt presentations were rated higher than audio-only presentations. On reduced numbers of test items, music undergraduate students, and piano-major observers rated excerpts higher than graduate students and faculty members, or non-piano-major observers, respectively. Whereas piano-major observers rated excerpts similarly across audio-only and audio-visual conditions, non-piano-major observers rated audio-only significantly lower than audio-visual presentations. The style and tempo of excerpts appeared to affect ratings differently according to level, and type of music training. On a few test items, piano-majors rated fast excerpts lower than non-piano-major observers, and excerpts of music from the classical period lower than excerpts of early 20th century Russian music. On two test items, graduate student and faculty observers rated fast excerpts lower than undergraduate student observers. Therefore, the ability to judge performance quality reliably appears to develop over time, and with opportunities to gain experience and develop the skill (Bergee, 1997). Results also suggest that observers who share the same type of specialist motor expertise as of the performer, or perhaps are more familiar with the music repertoire, might process sensory information slightly differently to other musically trained observers.

Thompson and Williamon (2003) asked three experienced evaluators to assess video-recorded tertiary-level student performances, using marking criteria based on an established assessment scheme in the UK. They found that there was some bias toward the evaluator’s own instrument. Specifically, the string specialist rated performances from the string group significantly lower than the other non-string-playing expert evaluators. However, the observation is somewhat contrary to a finding of Wapnick et al. (2004), who found that on some items, piano-majors gave significantly higher ratings than non-piano-major observers. Differences in results between the studies could be due to differences in level and type of observers’ music expertise. Alternatively, they might reflect issues with either or both of the different in the assessment schemes used.

Wapnick et al. (2004) reported that although the average reliability coefficient was significant, it was moderate at best. That reliability did not significantly differ between levels or type of music expertise indicates that perhaps such effects might emerge through responses made to intact, longer performances, rather than excerpts of performance. However, Thompson and Williamon (2003) did ask observers to rate intact performances, and observed only moderate inter-judge correlations. They also commented that the assessment criteria were very limited. Commonly used music performance assessment schemes employ rating dimensions such as in Thompson and Williamon (2003) and Wapnick et al. (2004). Therefore, there is a potential problem that issues of reliability, validity, and consistency in their use impacts on the ability to interpret results confidently.

Wrigley and Emmerson (2013) took an ecological approach to developing and evaluating a music performance assessment tool – the performance evaluation report (PERS). Although bringing some objectivity to a highly subjective task, the authors concluded that an element of subjectivity remained when judging music performance. Wrigley and Emmerson (2013) noted the challenge of developing a reliable and valid assessment scheme for performance evaluation of all instruments, because of instrument-specific differences. Thus, a further impediment could be that musicians apply any music performance assessment scheme in rather individual ways according to their own music, and instrument specific knowledge and motor expertise. Furthermore, none of the assessment schemes referred to here (Bergee, 1997; Thompson and Williamon, 2003; Wapnick et al., 2004; Wrigley and Emmerson, 2013) mentions aspects such as stage behavior and dress (Wapnick et al., 1998), or expressive bodily behavior (e.g., Davidson, 1993; Broughton and Stevens, 2009a), which have been shown to affect judgments of performance. These attributes could be tied up in many of the rating dimensions, and unaware to the judges themselves, may affect their evaluations of performances.

In sum, expert observers’ music experiences, and instrument-specific knowledge and motor expertise potentially shape the way they judge performance. However, there needs to be some evidence that the specific assessment scheme used to evaluate performance is reliable and valid in order to compare observers’ responses with some confidence.

The present study focuses on embodied expression, as a facet of performance quality, and uses effort-shape analysis as the assessment scheme. The effort-shape analysis system has previously been subjected to inter-judge reliability assessment (Broughton and Stevens, 2012). The present study not only uses this analytical system, but also generates new data to add to the evaluation of its efficacy as a tool for analyzing musicians’ expressive bodily behaviors. Expressiveness is often an integral rating item in the assessment of music performance quality (Thompson and Williamon, 2003; Wapnick et al., 2004; Wrigley and Emmerson, 2013). Indeed, expressivity is argued to be an important facet of engagement with music (Maes et al., 2014). As evidence shows musicians’ expressive bodily behaviors affect judgments of performance, the present study focuses on *expressive moments* perceived in audio-visual music performance presentations. These are any moments in audio-visual presentation of intact performance that stand out to the observer as expressive in some way. This acknowledges that at any point judgments might be dominated either by vision, sound, or a combination of both.

The present study also extends work in the area of performance quality assessment by providing information as to *how* observers’ perception of performance evolves over the course of the performance. The effort-shape analysis system as applied here, which will be explained later, requests observers to document any expressive moment they perceive at the point in the performance when they perceive it. This is in stark contrast to the usual method where observers assess the performance by making single retrospective judgments on a number of criteria. The effort-shape analysis system is not likely to replace current methods of performance assessment. However, its use in the present study will contribute information on how individual differences might contribute to different observers’ perception of expressive performance, which may be useful to consider in concert with usual performance assessment methods (Thompson and Williamon, 2003).

The idea of individual embodied experiences shaping perception and judgments of performance is supported by theory and evidence for embodied cognition.

### EMBODIED COGNITION: ACTION AND MOTOR EXPERTISE EFFECTS ON ACTION PERCEPTION

Historically, cognition was believed to operate on amodal symbols as representations of knowledge and experience in semantic memory. But an emerging theory of cognition is that of the “embodied” or “grounded” view, which proposes that modal simulations, situated action, and bodily states underpin mental processing (Barsalou, 2008; Shapiro, 2011). While various aspects of an embodied, or grounded cognitive perspective have been debated (e.g., Wilson, 2002; Shapiro, 2007), mounting evidence suggests robust links between perception and action exist, that are shaped by our interactions with the environment. Thus, perception–action couplings form important components of a dynamical system (van Gelder, 1995), in which the body and environment are integral to cognition (Wilson and Golonka, 2013).

Engaging with the external environment involves sensory and motor components, which give rise to internal models about body-environment relationships (Wolpert et al., 1995). Internal models can be of two types: inverse or forward. Inverse models of perception and action use incoming sensory information to provide motor commands to affect a desired change in state. Forward models of perception and action are predictive in that they permit prediction of the potential outcome a motor command will have on the body or environment. In contrast to the inverse model, in the forward model action flows to perception. Forward and inverse models are intricately linked in perception and action whether movements are executed, experienced, or just observed (Wolpert and Kawato, 1998; Maes et al., 2014). However, Halász and Cunnington (2012) argue that predictive theories, based on forward models, offer the best explanation for how the motor system shapes action perception. In the following section we outline behavioral and neuroscience studies providing evidence for inverse and forward models of perception and action.

As evidence for an inverse model of perception and action, Tucker and Ellis (1998) observed that reaction times responding to an object were faster when made by the hand that would be used to grasp the object. The left hand was faster to respond to an object with the handle of the left in a picture, whereas the right hand was faster to respond when the handle was on the right of the pictured object. Thus, perception automatically prompted an appropriate action plan to achieve a physical goal. Behavior-based music studies demonstrated that motor reaction time in response to sounds were faster for participants possessing considerable experience of specific sound–action pairings, (Trimarchi and Luzzatti, 2011; Stewart et al., 2013). In a study of piano practice using electro-encephalography (EEG), Bangert and Altenmüller (2003) found evidence to suggest that auditory-sensorimotor integration emerges in minutes, and is established within weeks of commencing learning. Thus, experiences with visuo-motor, or audio-motor pairings facilitate fast motor responses to like perceptual stimuli. Evidence for a *mirror neuron system* (MNS) further supports the inverse model of perception and action by demonstrating that the observation of action can elicit neural activation as if producing the observed action.

At a neural level, the discovery of mirror neurons in the monkey’s premotor cortex (Di Pellegrino et al., 1992; Gallese et al., 1996) showed that the same cells were activated for action perception as they were for action production. Studies with human participants have demonstrated a MNS response when observing a range of actions, including hand movements (Decety et al., 1997) and reaching actions (Filimon et al., 2007), through to learned dance movements in an experimental setting (Cross et al., 2009). Specialist expertise, or training has been shown to modify the MNS response to action stimuli as observed in music (piano playing, Haslinger et al., 2005), and artistic movement (classical ballet and capoeira – an Afro-Brazilian martial arts-dance fusion, Calvo-Merino et al., 2005) contexts. Even imagining performing a previously executed action elicits some neural activation as if producing the action (Filimon et al., 2007; Donoghue, 2008).

The hypothesized human MNS has been theorized as a key mechanism in facilitating action understanding and underpinning human communicative processes (Gallese and Goldman, 1998; Hommel et al., 2001; Jackson and Decety, 2004; Rizzolatti and Craighero, 2004). Furthermore, in the case of expressive action and of direct relevance to the present study, imitating, rather than merely observing, has been shown to elicit patterns of neural responses associated with emotional empathy (Carr et al., 2003). With respect to music, recent suggestions have been made that musical communication involves expressive motor co-representations, shared between performer and perceiver, elicited through auditory as well as visual information (Molnar-Szakacs and Overy, 2006; Leman, 2008; Overy and Molnar-Szakacs, 2009). However, it is plausible that individual differences in motor expertise, audio-motor, and visuo-motor couplings shape such representations.

Evidence for forward models, whereby action flows to perception, is found in studies demonstrating that movement training, or even the experience of movement, influences perception. For example, Hecht et al. (2001) observed a transfer effect from action to perception. Training to produce simple timed arm movements facilitated more accurate and less variable judgments of similarly timed visual movement patterns. Furthermore, action–perception transfer (APT) was not dependent on planning and executing the arm movement. Kinesthetic feedback from passive movement of the arm was sufficient to create APT. Active and passive movement to the beat has been shown to improve listeners’ perception of timing (Manning and Schutz, 2013), metric structure (Phillips-Silver and Trainor, 2007, 2008), and pulse (Su and Pöppel, 2012). Maes and Leman (2013) found that children’s movement experience performing happy or sad choreography with emotionally ambiguous music affected their perception of the musical expression. Therefore, planned and executed movement, or even just kinesthetic feedback from passive movement experience can mold perception in ways such as helping disambiguate information, direct attention to cues, and affect esthetic judgments.

Maes et al. (2014) argue that through sensory-motor associative learning processes, music and actions become integrated along with related sensory and affective states. The authors draw on Heyes’s (2010) associative hypothesis, which links MNS development to sensory-motor associative learning. Repeated experiences bind sensory and motor information together to form internal models. Inverse models automatically activate a motor representation from perception of sensory information. Forward models automatically activate a sensory representation stemming from planned or executed motor acts. However, the specific features of the learning process (e.g., long-term instrumental music training vs. training in an experiment session) might indicate different types, strengths, and durations for the retention of associative learning effects. An effect associated with long-term instrumental music training might be expert performance.

Expertise in music is characterized by superior performance, facilitated by specialized mental and physiological representations, developed over a long period (about 10 years) of sustained deliberate practice (Ericsson et al., 1993; Ericsson and Towne, 2010). Research with expert performers in other fields, such as aviation, suggests that experience impacts not only on perception, but also on cognitive decision-making processes. For example, it has been suggested that task-specific experience develops pilots’ structured mental models of the task environment in long-term memory (Doane et al., 2004), which directs their attention (Bellenkes et al., 1997; Schriver et al., 2008) when performing a task and making decisions in the current environment. Task-specific experience, rather than general experience in a field is important in decision-making processes (Ericsson, 2006). Therefore, individuals who might be similarly considered as music expert performers could have task-specific experience playing or assessing different instruments or voice types. Thus, diversity of individual experiences potentially affects how each music expert performer perceives, and judges music performance.

In the present study, expert musicians, possessing considerable task-specific experience as performers and performance assessors, acted as participants. The participants differed in specific motor expertise. A percussionist and a singer analyzed the performance of another percussionist, who conducted an analysis of her own performance. Only the performing percussionist was familiar with the particular music piece serving as stimuli. Based on the preceding, it is plausible that individual differences in observer specialist motor expertise influence decisions arrived at in the course of analyzing performing musicians’ expressive bodily behaviors.

An inter-judge reliability study investigated effort-shape analysis as a system of analyzing musicians’ expressive bodily behaviors (Broughton and Stevens, 2012). Results suggested that professional musicians’ reliable implementation of a component of the system required specialist motor expertise, or embodied knowledge, of the same type as the instrument being performed and being observed. Other components of the system requiring general experience of expressive bodily action were implemented reliably. Effort-shape analysis operates on visual perception of expressive action, as well as kinesthetic, or internal feedback from imitating or covertly simulating expressive actions. Therefore, it draws on forward and inverse models of perception and action. Additionally, processes of introspection (Barsalou, 2008, 2009) are crucial to analyze and understand the performer’s internal states – affect and intentions – as aspects inherent to the expressive action observed. We would expect participants’ analyses to vary for components of the system requiring understanding of marimba-playing actions. However, we would expect participants’ analyses to be similar for components of the system drawing on more general embodied experience of human expressive bodily actions.

Given the contextual literature and proposed outcomes of a study of musical movement judgment, it is important to overview a system to analyze musicians’ expressive bodily behaviors. Given familiarity with and demonstrated effectiveness of the system, we have chosen Laban Movement Analysis (LMA), and specifically, *effort-shape analysis*.

### LABAN EFFORT-SHAPE ANALYSIS

Laban Movement Analysis encompasses a range of systematic approaches to analysing and understanding action. Unifying all is the principle that observable movement reflects the individual’s inner motivation for movement (Bartenieff and Lewis, 1980), and the techniques used to observe and analyze action. LMA proposes an embodied approach to perception and cognition of human movement behavior. As well as using vision, the LMA observational approach draws on the observers’ subjective kinesthetic, embodied experiences arising through the physical experience of movement (Bartenieff and Lewis, 1980). LMA has been used in a range of settings to analyze and document human movement behavior. For example, the LMA approach has been applied to analyze expressive movement in dance (Bartenieff et al., 1984; Hutchinson Guest, 2005), music (Broughton and Stevens, 2012), anthropological (Jablonko and Kagan, 1988), and clinical (Higgins, 1993; Foroud and Whishaw, 2006) contexts. The LMA approach considers four main components: the *body* moving through *space* results in various *shapes*, and bodily movement requires *effort* to some degree (Bartenieff and Lewis, 1980). The expressive qualities of dynamic movement are captured in effort and shape components. Effort-shape analysis focuses on these components to analyze expressive qualities in movement, that is, how rather than what movement is performed (Davis, 1970). The present paper applies the effort-shape system to analyze expressive qualities perceived in a marimba player’s performance.

**FIGURE 1:**
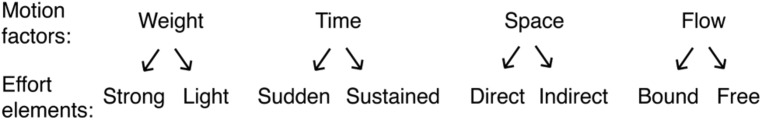
**The four motion factors and their associated effort elements – the primary components of effort-shape analysis**.

Effort-shape analysis draws on LMA visual and kinesthetic observational techniques to understand expressive qualities of human movement behavior. A full description of the effort-shape analytical system can be found in Broughton and Stevens (2012). Here we offer a description that outlines the key features of the system. Firstly, we discuss the concept of effort. In expressive human movement effort is recognizable in the patterns of tension, release and phrasing of physical exertion (Maletic, 1987). Effortful, expressive human movement can be analyzed in terms of combinations of different *effort elements* associated with *motion factors* (see **Figure 1**). Different effort element combinations reveal different expressive qualities in human movement (Maletic, 1987).

Expressive action that is most obvious involves three of the four motion factors (Bartenieff and Lewis, 1980). Different combinations of effort elements associated with the motion factors weight, time, and space result in eight *basic effort actions* (see **Table 1**). Replacing the motion factor of weight, time, or space with flow results, in one of three *transformation drives*. For the eight basic effort actions and three transformation drives, the metaphoric name refers to the visual appearance, and kinesthetic experience of performing the action. The eight basic effort actions are “goal-directed,” essential working actions (Laban and Lawrence, 1974). That is, each action described by the metaphoric name features two distinct phases – exertion and relaxation. Transformation drives can appear as “non-goal-directed” actions. Movement or stillness might be highly expressive, yet not directed toward a specific physical goal. For example, “spell” can appear as movement or stillness, as if the person is casting a spell. “Vision” has a disembodied quality as if the person is deep in thought or concentrating. “Passion” can appear as enraged gesticulations, or those of tenderness.

**Table 1:** The metaphoric names for the eight basic effort actions, three transformation drives, and their constituent motion factors and effort elements.

	Metaphoric name	Weight	Space	Time	Flow
Basic effort actions “goal-directed” movements	“Punch”	Strong	Direct	Sudden	na
	“Dab”	Light	Direct	Sudden	na
	“Press”	Strong	Direct	Sustained	na
	“Glide”	Light	Direct	Sustained	na
	“Slash”	Strong	Indirect	Sudden	na
	“Flick”	Light	Indirect	Sudden	na
	“Wring”	Strong	Indirect	Sustained	na
	“Float”	Light	Indirect	Sustained	na
Transformation drives “non-goal-directed” movements	“Passion”	Strong/Light	na	Sudden/Sustained	Bound/Free
	“Spell”	Strong/Light	Direct/Indirect	na	Bound/Free
	“Vision”	na	Direct/Indirect	Sudden/Sustained	Bound/Free

Analysis of effort in bodily movement or stillness that stands out to the observer as expressive, initially involves a matching process. Based on the visual appearance of the expressive action, and the subjective experience arising from overtly or covertly performing the action, the observer selects the basic effort action or transformation drive that best fits their perceptual experience. In many instances, the metaphoric name is enough on which to base the analysis. In some instances, though, the observer may have to take a “bottom up” approach to analyze effort. That is, build their analysis on whether they perceive the action as “goal-directed”, or “non-goal-directed”, and make decisions about the constituent motion factors and effort elements they perceive in the action. While *gestural effort* refers to the use of only the body part necessary to perform the job, *postural effort* indicates involvement of the whole body in the activity (Lamb and Watson, 1979; Bartenieff and Lewis, 1980). Therefore, the way the body takes shape in space is linked to expressive, effortful action (Royce, 2002).

**FIGURE 2:**
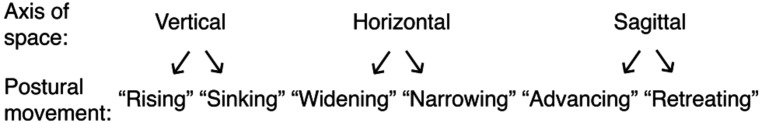
**Postural shaping movement terms associate with the vertical, horizontal, and sagittal axes of space**.

**FIGURE 3:**
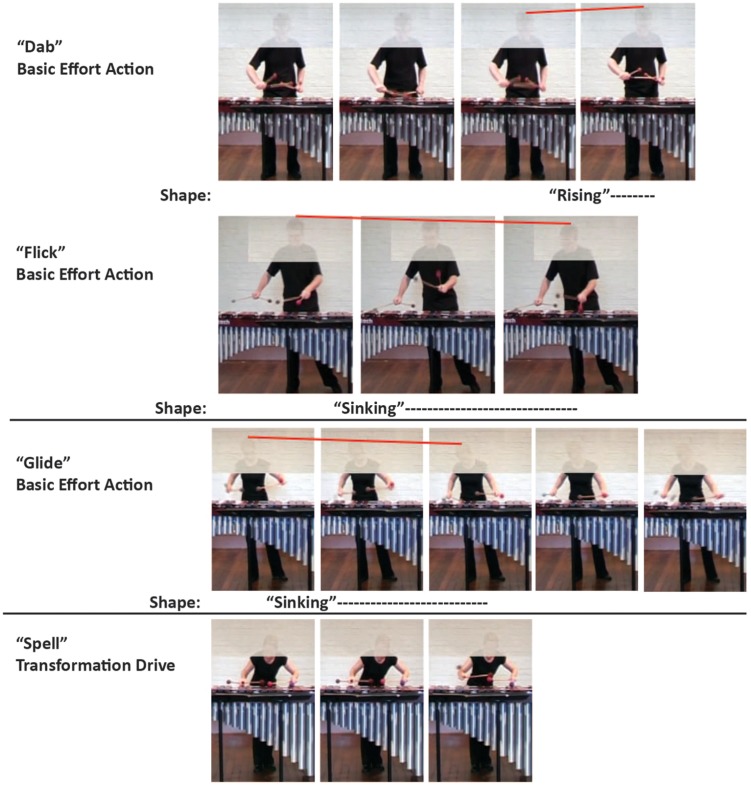
**A few expressive effort and shape feature movement examples illustrated through series of still images.** The images and accompanying analyses are taken from participants’ analyses in Broughton and Stevens (2012). The examples from that inter-judge reliability study are representative of agreement on action type and location between participants. The red line above the performers’ heads in the basic effort action frames shows the direction of movement.

Shape refers to bodily movement along the vertical, horizontal, and sagittal axes of space (see **Figure 2**). Shape features are analyzed as the body’s postural movement along the axes of space that the observer perceives as involved in effortful, expressive bodily activity: “rising”/“sinking” (vertical), “widening”/“narrowing” (horizontal), “advancing”/“retreating” (sagittal). **Figure 3** provides illustrative examples of a few expressive effort actions and shape features through a series of still images drawn from participants’ analyses in Broughton and Stevens (2012) inter-judge reliability study. It is important to note that on many dimensions, the observable differences are subtle, but were reliably identified by judges. Effort and shape analyses can then be documented as overall frequency counts, indicating overall patterns of bodily behavior, or in the temporal domain. The specific locations at which effort and shape features are observed can be identified either on the music score, or specific points in audio-visual recordings of performance. Utilizing the richness of the Laban system, the present study analyzes observers’ effort-shape data in frequency and temporal domains, from an audio-visual recording of a marimba player in recital. Temporal data about effort and shape observations take the form of the time stamp in the audio-visual recording, and duration of participants’ observations.

A few studies have reported inter-judge reliability for various effort (McCoubrey, unpublished) and shape (Davis, 1987; Du Nann Winter et al., 1989) concepts and components, and higher-order movement analysis systems incorporating effort and shape components (Davis, 1983; Sossin, 1987). Broughton and Stevens (2012) found that following training, participants implemented the effort-shape analytical system as described here and applied to marimba performance reliably for transformation drives and shape features, but not basic effort actions. The authors concluded that participants’ lack of specialist marimba-playing motor expertise might have impacted their ability to analyze expressive marimba-playing actions. The present study includes as participants an expert marimba playing percussionist, and an expert singer (who is inexperienced with percussion and has not played the marimba) to re-investigate inter-judge reliability for basic effort actions.

As effort-shape analysis draws on processes of visual and kinesthetic movement imagery, participants in the present study were screened for sound and comparable functioning in this ability using an established test – the Movement Imagery Questionnaire-Revised (MIQ-R, Hall and Martin, 1997). Additionally, participants were screened for sound and comparable interpersonal non-verbal sensitivity through a test of the ability to identify another’s socio-emotional attitude through non-verbal channels – the short version of the Profile of Non-verbal Sensitivity (PONS, Rosenthal et al., 1979), which is the MiniPONS (Bänziger et al., 2011). To the authors’ knowledge, this is the first time the miniPONS has been used within a music performance research context. The MIQ-R has been used once previously in a study of professional and undergraduate solo marimba players’ strategies and imagery used in practice (Broughton and Stevens, 2009b).

### RESEARCH AIM, DESIGN, AND HYPOTHESIS

The aim of the present study was to investigate to what degree a marimbist’s effort-shape analysis of her own expressive bodily behaviors is reflected in the effort-shape analyses conducted by expert musician observers who differ in marimba-playing motor expertise, and familiarity with the music composition. The design was an observational study involving an independent analysis task. We hypothesized that following training in the effort-shape analytical system, observers’ independently conducted effort-shape analyses differ according to marimba-playing motor expertise, and familiarity with the music performed.

## MATERIALS AND METHODS

### PARTICIPANTS

Participants were two professional percussionists and experienced marimba players, and one professional classical singer. One of the professional percussionists was also the performer of the audio-visual music material for analysis, and is the first author of the present paper. The *first author* (aged 35 years), other *percussionist* (aged 29 years), and *singer* (aged 49 years) had completed at least one degree program in music, and had performing experience at national and international levels. While the performer (first author) and percussionist observer shared comparable embodied marimba-playing expertise, the singer had never played the marimba or indeed any percussion instruments in any serious manner before.

### MATERIALS

The first author provided an audio-visual excerpt of herself playing marimba in duet with a flutist, which was recorded during a professional music chamber recital. The material for analysis was the fourth movement of *Cinq Pantomimes Pour Flute et Marimba by* Damase (2002; 1 min 33 s duration). Only the first author’s marimba performance was analyzed for the purposes of the present study. This work was unfamiliar to the percussionist and singer observers. Participants were provided a digital audio-visual recording of the performance excerpt for analysis, playable on television or computer, with a copy of the music score. Participants also received written instructions explaining the effort-shape analytical system, and audio-visual training examples of marimba performance accompanied with the music scores for each example. The training examples were drawn from Broughton and Stevens (2012). Only examples in which the four analyst participants from the previous study agreed about the categorization of a particular expressive bodily behavior observed at the same temporal location were used as training examples in the present study.

Participants documented their effort-shape analysis using *ELAN* (version 4.5.0) software for annotating audio-visual material. ELAN is freely available from Max Planck Institute for Psycholinguistics, The Language Archive, Nijmegen, The Netherlands^1^. ELAN has previously been used to annotate human gestural behavior (Lausberg and Sloetjes, 2009; Sassenberg et al., 2011).

Participants completed two tests assessing individual differences in abilities relevant to the independent effort-shape analysis task. First, participants completed the MiniPONS test, which assesses non-verbal sensitivity as related to socio-emotional competency (Bänziger et al., 2011). It is a short version of the established PONS (Rosenthal et al., 1979), designed to assess individual differences in socio-emotional competency (emotion recognition, communicating intentions, and interpersonal attitudes) through different non-verbal modalities. Recent research suggests that the MiniPONS loads on to emotional abilities in the domain of social and emotional effectiveness constructs (SEECs; Schlegel et al., 2013).

The MiniPONS is a self-report, multichannel test comprising 64 items. Participants are presented with 2-s non-verbal clips (edited audio-only, audio-visual, or visual-only) drawn from longer videos of a woman expressing a variety of emotional qualities, seemingly engaged in different interpersonal interactions. The task is one of alternate forced choice. The participant is asked to choose the best fitting situational explanation to the expression presented. Scores range from zero (no correct responses) to 64 (all correct responses). Bänziger et al. (2011) report satisfactory validity for the MiniPONS when total results were correlated with the full PONS test (*r* = 0.70), and other established emotional recognition tests. Internal consistency as measured by intraclass correlation coefficients (ICC) was sound (single item ICC = 0.021, combined items ICC = 0.566). According to Rosenthal and Rosnow (2008), combined items ICCs are analogous to Cronbach’s Alpha, which measures the internal consistency of the complete scale or subscale. Test–retest reliability for the 64 items of the full PONS that construe the MiniPONS was also satisfactory (*r* = 0.64). The MiniPONS can be accessed online^2^.

The second test that participants completed was the MIQ-R (Hall and Martin, 1997). The MIQ-R is a revised version of the self-report MIQ (Hall and Pongrac, 1983). Essentially, the revision is the dropping of 10 items to reduce the 18-item MIQ to an eight-item test (MIQ-R). The MIQ-R assesses movement imagery ability on two subscales – visual and kinesthetic. Each item requests the participant to perform a simple physical action. Then the participant is asked to imagine seeing (visual subscale) or feeling (kinesthetic subscale) themself performing that same action, and rate the ease/difficulty with which they could perform the mental task on a seven-point Likert scale. Scores are summed separately for items on the visual and kinesthetic subscales. Scores can range from 4 to 28; higher scores indicate greater ease of imagery. Internal consistency coefficients above 0.79 for both visual and kinesthetic MIQ-R subscales have been reported (Vadoa et al., 1997; Monsma et al., 2009). Test–retest reliability (0.80 for visual and 0.81 for kinesthetic subscales) is sound (Monsma et al., 2009). The MIQ-R’s bi-factorial structure has also been confirmed (Lorant and Nicolas, 2004; Monsma et al., 2009).

### PROCEDURE

Participants gave written informed consent prior to taking part in the study. The study conformed to Australian regulatory standards. The University of Western Sydney Human Research Ethics Committee approved the original observational study methodology. Further approval from the University of Western Australia Human Research Ethics Officer was obtained when the first author moved institutions. Being experienced in conducting effort-shape analysis in the manner necessary to the study and training others to implement the system applied to marimba performance (see Broughton and Stevens, 2012), the first author provided a training session to the professional percussionist/expert marimba player and classical singer observers. At the time the study was conducted, there was no other individual suitably experienced in the analytical system and its application to music performance to provide the training. During an individual one-and-a-half hour training session, each participant was introduced to the effort-shape analytical system, practiced the actions and observational techniques, provided with written instructions (see Broughton and Stevens, 2012), and audio-visual examples of marimba performance illustrating the various components of the system. The audio-visual training examples were drawn from the results of Broughton and Stevens (2012) where all observer participants in that study agreed on the same type of expressive action observed at the same time. Participants also physically practiced the subcomponents of the system to understand the visual appearance and kinesthetic experience of the bodily actions. Once satisfied they understood the effort-shape analytical system and how to apply it to marimba performance, the participants were instructed on the procedure of analyzing the performance material, and shown how to document their analyses using ELAN software.

To conduct the independent analysis task, the participants were instructed to first note each moment that they perceived as expressive (expressive moment) in the audio-visual performance material. Once expressive moments were identified, participants analyzed those moments in detail. At each expressive moment, participants documented in ELAN, their perception of basic effort action(s) or transformation drive(s), and/or shape feature(s) observed at the time point and for the duration they perceived. The first author, percussionist, and singer then independently analyzed the audio-visual performance material at their convenience, over a period of approximately 1 week. After first noting the expressive moments in the audio-visual performance material played in real time, participants were free to start and stop the recording at will, and analyze with and without sound, as they deemed necessary during the analytical process. Participants were afforded these freedoms since participants in McCoubrey’s (unpublished) investigation, examining reliability for motion factors and effort elements using cello performance, expressed frustration at not being able to review the film clip at will. In another system incorporating effort and shape concepts, participants were able to review the footage at will (Davis, 1983). Participants in Broughton and Stevens (2012) study were permitted to review the audio-visual material in the manner reported in the present study. Diarising the time spent on the analyses, and how much was done without sound was not requested of participants for the present paper. However, during debriefing, participants reported that the task took approximately 4–5 h to complete. During this time, participants also completed the MIQ-R (Hall and Martin, 1997) as a pen-and-paper questionnaire, and the MiniPONS (Bänziger et al., 2011) online^3^.

Each participant’s effort-shape analysis was exported from ELAN as a text file documenting their perception of expressive moments categorized into basic effort actions, transformation drives, and/or shape features, as well as the start time and the duration of each component observed.

## RESULTS

Participant’s scores on the MiniPONS (Bänziger et al., 2011) were comparable, and accuracy detection was well above chance: first author, 53 (of 64) correct (83%); percussionist, 52 correct (81%); singer, 51 correct (80%). Participants also reported comparable and high visual [first author score = 25 (scale range 4–28), percussionist score = 27, singer score = 27] and kinesthetic (first author score = 25, percussionist score = 25, singer score = 26) movement imagery ability as measured using the self-report MIQ-R (Hall and Martin, 1997). All participants self-reported sound socio-emotional competency and movement imagery ability indicating they would be conducting the independent analysis task from a “level playing field.”

Separate analyses compare participants’ observations for basic effort actions, transformation drives, and shape features. Results of analyses based on frequency of observation data are reported first. These results are followed by analyses based on temporal location and duration of observations.

### FREQUENCY OF OBSERVATIONS

**FIGURE 4:**
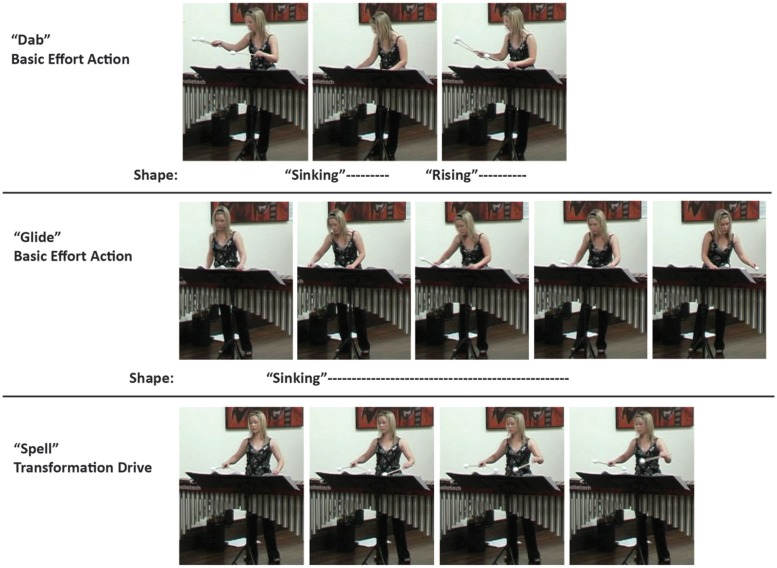
**This series of still images illustrates movement examples of expressive effort and shape features drawn from the performance recording**.

A chi-square goodness-of-fit test showed significant differences between participants’ frequency of observations for basic effort actions, χ^2^(2, *N* = 134) = 11.39, *p* < 0.01. The first author observed the most basic effort actions (58, 43.28%), followed by the percussionist (49, 36.57%), then the singer (27, 20.15%). Given the closeness of observational frequencies found, follow-up tests were conducted. With a Bonferroni adjusted alpha level for multiple comparisons (α = 0.016), results revealed that the first author and percussionist participant did not significantly differ in their frequency of basic effort action observations, χ^2^(1, *N* = 107) = 0.76, *p* = 0.384. All other comparisons between participants were significantly different: first author and singer, χ^2^(1, *N* = 85) = 11.31, *p* < 0.01; percussionist and singer, χ^2^(1, *N* = 76) = 6.37, *p* < 0.015. All three participants observed “dab” and “glide” basic effort actions, and the first author and percussionist observed “flick” and “float” additionally. The first author also documented a few “punch” basic effort actions. “Press,” “slash,” and “wring” were not reported. **Figure 4** provides some illustrative examples of effort and shape features through a series of still images drawn from the performance recording.

Since the singer did not observe any transformation drives, a chi-square goodness-of-fit test only compared frequency of observations between the first author and percussionist. This test yielded a significant difference, χ^2^(1, *N* = 26) = 15.39, *p* < 0.001. The first author observed more transformation drives (23, 88.46%), than the percussionist (3, 11.54%). The first author and percussionist observed a few “spell” transformation drive, but only the first author reported a couple of “passion” and several “vision.”

A chi-square goodness-of-fit test revealed significant differences between participants’ frequency of observations for shape features, χ^2^(2, *N* = 150) = 70.68, *p* < 0.001. The first author observed the most shape features (91, 60.67%), followed by the singer (52, 34.67%), then the percussionist (7, 4.67%). Given the disparity of observational frequencies found, follow-up tests were deemed unnecessary. All three participants observed a “rising” shape feature. The first author and percussionist reported “widening” and “retreating,” and the first author and singer reported “sinking” shape features additionally. Only the first author reported “narrowing” and “advancing” shape features.

### SHARED TEMPORAL LOCATION AND DURATION OF OBSERVATIONS

**FIGURE 5:**
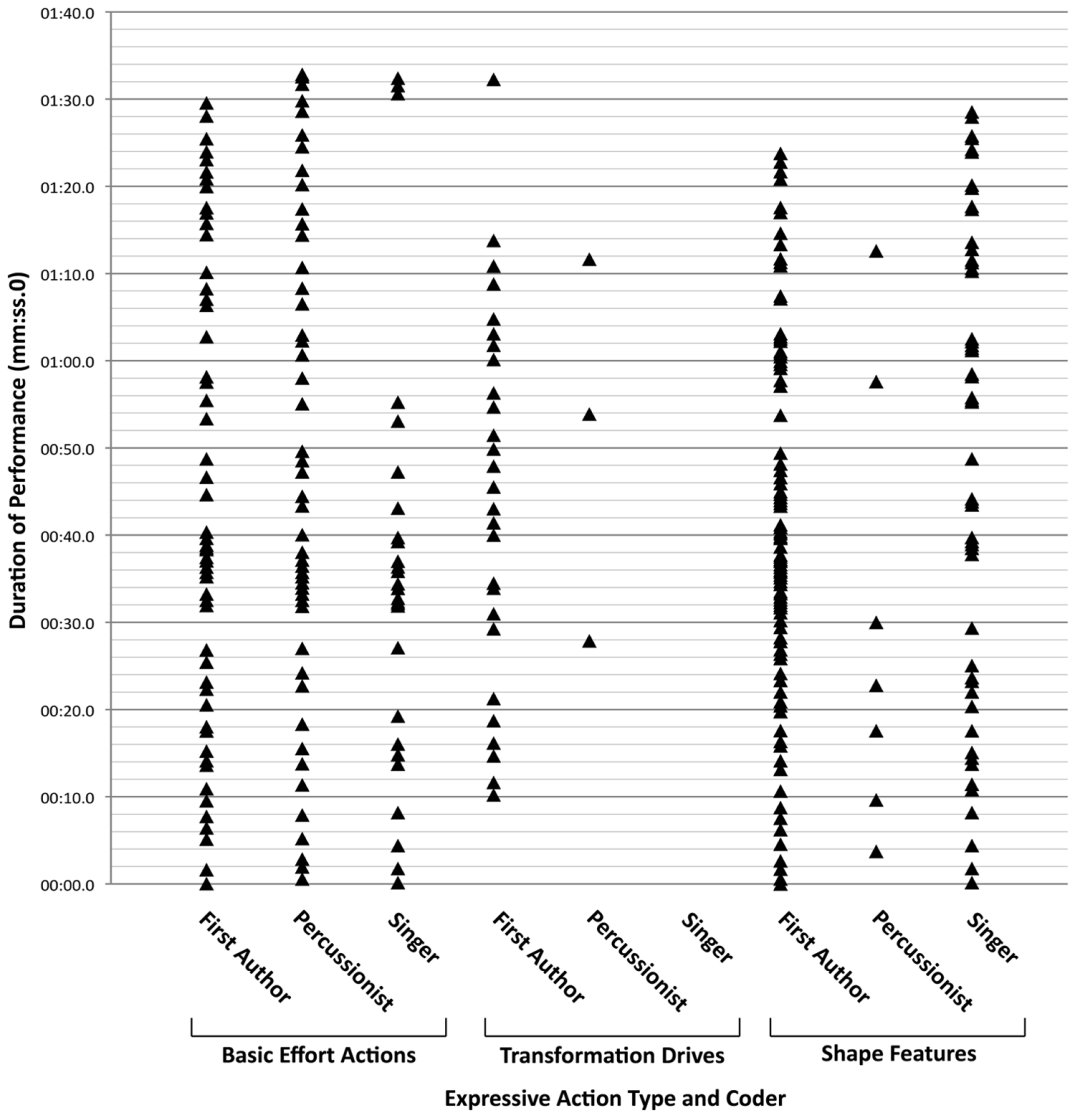
**The distribution of participants’ effort-shape observations across the duration of the performance stimulus material.** The minor gridlines delineate the two-second bins that were examined for agreement between participants for the same action type in each category (basic effort actions, transformation drives, and shape features).

To ascertain whether or not participants were making similar effort and shape observations at similar times involved “binning” the performance into brief, 2-s intervals. This resulted in a total of 47 *bins*. **Figure 5** shows the distribution of participants’ basic effort action, transformation drive, and shape feature observations across the duration of the music performance stimulus material. Each 2-s bin was examined across all participants for agreement in observation of the same types of effort or shape components. Where at least two participants documented observing the same type of basic effort action, transformation drive, or shape feature in a bin, agreement was expressed proportionally. That is, the number of same-type observations that were shared with another or both participants relative to the total number of observations for that category (basic effort actions, transformation drives, or shape features) for each bin, Each of the 47 bins had separate agreement proportions for basic effort actions, transformation drives, and shape features.

There was 55.36% (SD = 40.87%) mean agreement between participants for basic effort actions (two participants agreed in 44 bins, three agreed in four bins). Transformation drives were observed in 23 bins, but there was no agreement between participants. Therefore, transformation drives were excluded from further analyses of bins. There was 40.63% (SD = 41.06%) mean agreement between participants for shape features (two participants agreed in 42 bins, three agreed in five bins). To provide some context in which to view these results, we created comparison distributions from the data. We took this approach, as there is only one published study that has applied effort-shape analysis to music performance in this manner (Broughton and Stevens, 2012), and whose research purpose and music stimulus material were different to the present investigation. In the present study, we randomly re-distributed each participant’s observations across the performance, and then re-examined the 2-s bins for agreement between participants. We performed this procedure three times, and averaged the obtained binned percentage agreement between participants across the three instances. We took this approach, as it was not possible to ascertain binned agreement between participants for each of many random distributions created from the data using traditional bootstrapping procedures. There was 23.72% (SD = 20.62%) mean agreement for randomly distributed basic effort action observations (two participants agreed on average in 44 bins, three agreed in three bins), and 29.78% (SD = 19.18%) mean agreement for shape features (two participants agreed on average in 45 bins, three agreed in two bins). There was no correlation between the actual and randomly distributed observations for basic effort actions, *r* = -0.20, *n* = 47, *p* = 0.17, or shape features, *r* = -0.03, *n* = 47, *p* = 0.85.

Dependent *t*-tests were conducted on the agreement percentage between actual and randomly distributed observations for basic effort actions, transformation drives, and shape features separately. Normality assumptions were violated, however, considering the sample size, the tests were considered robust. Results indicated a statistically significant difference between the actual and random observations for basic effort actions, *t*(46) = 4.39, *p* < 0.01. The mean percentage agreement was higher for the actual than the randomly distributed observations. Statistical significance was not achieved for shape feature *t*(46) = 1.62, *p* = 0.11 observations.

Though a useful starting point, the binning method segments the performance material in an insensitive manner. It does not take into account the fact that different expressive bodily behaviors have different durations, which might extend across bins. For example, a transformation drive “spell”-like movement might take longer to perform in comparison to a basic effort action “dab” action, which is over and done with quite quickly. Furthermore, the binning method could over report agreement of shorter-duration expressive actions. For example, the same type of observation reported at the beginning of a bin by one participant, and the end by another, could be considered as agreement on single action, when in reality there are two different expressive moments of short duration – one reported by one participant, and the other by another participant. In addition, because basic effort actions are “goal-directed” movements, with distinct tension phrasings of exertion and release, it is likely that participants would notice and report the onset of the action with low temporal variability. By contrast, it might be more difficult to pinpoint the exact onset of a transformation drive being “non-goal-directed” action, expressive of mood, and lacking clear exertion-release tension phrasings in comparison to basic effort actions. The durations and onsets of shape features would be expected to vary, taking on the same characteristics as the different types of effortful actions with which they are allied.

We embarked on an additional type of analysis, setting different criteria for the temporal onset of basic effort action, transformation drive, and shape feature observations in order for locations of expressive observations to qualify as a shared. Without precedence in the literature for precise effort-shape action onset timings between participants or durations, we constructed our criteria based on description of effort-shape actions in the literature (e.g., Laban and Lawrence, 1974; Bartenieff and Lewis, 1980; Maletic, 1987; Laban, 1988). We then examined the data for confirmation of the adequacy of our criteria. For basic effort actions a criterion of “less than 1 s” was set. That is, where the start times of expressive observations documented by participants fell within a second of each other, the location was considered shared. A shared location could include only two, or all three participants. This criterion was deemed adequate when examination of the data revealed that the variability of basic effort action observation start times between participants was on average 0.3 s (SD = 0.3 s). The average duration of basic effort action observations was 0.7 s (SD = 0.2 s).

The criterion for shared observation location for transformation drives was extended to “less than 2.5 s.” This wider range was adopted since transformation drive expressive bodily behaviors evoke an overarching mood or quality and can be less tangible in appearance than basic effort actions, which feature two rhythmic phases of exertion and relaxation (Bartenieff and Lewis, 1980). Examination of the data revealed that the variability of transformation drive observation start times was on average 0.8 s (SD = 0.8 s). The average duration of transformation drive observations was 1.5 s (SD = 0.7 s).

There were six instances of shared observational locations where one participant reported a basic effort action, and another reported a transformation drive. In all of these cases, which were fairly evenly distributed over the performance duration, the first author reported the transformation drive (three “vision,” two “passion,” and one “spell”). These six instances were resolved as follows. In one location where two participants noted a basic effort action, and one participant a transformation drive observation, the basic effort action observation was adopted. In the remaining five observational locations that were shared between two participants, where one observed a basic effort action and the other a transformation drive, a process of random selection was employed. This resulted in four observations being categorized as transformation drives, and one as a basic effort action. These categorizations were then used in the analyses that followed.

**FIGURE 6:**
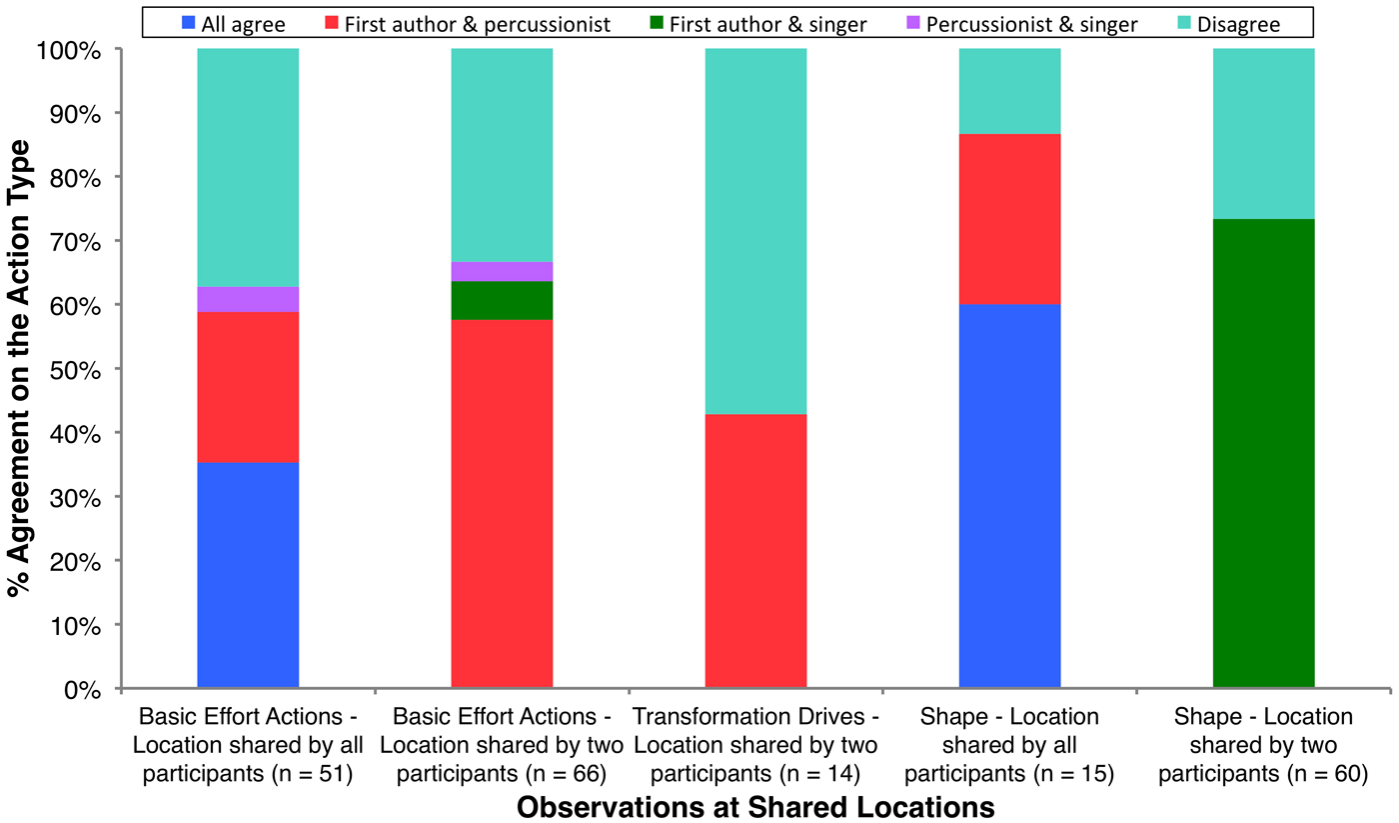
**Breakdown of agreement (and disagreement) between participants for the same type of action within each category (basic effort actions, transformation drives, and shape features) at the same temporal location.** For *n* locations shared by two or three participants, percentages of agreement and disagreement sum to 100% separately.

According to the criterion set for basic effort actions, there were 50 locations of shared observations. All three participants made observations at 17 (34.00%) of these shared locations, and two participants made observations in the remaining 33 (66.00%) locations. The 50 shared locations accounted for 117 (87.31%) of the total 134 basic effort action observations aggregated between participants. In 35 (70%) of the 50 shared locations, at least two participants reported the same basic effort action type at the same location. Observations of the same action type at the same location accounted for 76 (64.96%) of the total 117 basic effort action observations at shared locations. **Figure 6** shows the breakdown of action type agreement between participants at shared observational locations. At the locations where participants agreed that there was a basic effort action, but categorized it differently (“Disagree” portions of the two basic effort action columns in **Figure 6**), the majority of disagreements were due to differences in one effort elements. For example, “dab” and “glide” both share light weight, and direct space, but where “dab” exhibits a sudden approach to time, “glide” exhibits a sustained approach.

According to the criterion set for transformation drives, there were seven locations of shared observations. The first author and percussionist reported observing the same type of transformation drive (“spell”) at three (42.86%) of the seven shared locations. These three locations accounted for six of the total 14 transformation drive observations at shared locations (42.86%). The remaining four locations represented confusion between a basic effort action and a transformation drive (“Disagree” in the single transformation drive column in **Figure 6**). That is, at the location the first author reported a transformation drive; another participant reported a basic effort action.

The way the body takes shape in space is inextricably linked to effort, that is, there exist affinities between body, space, and effort (Bartenieff and Lewis, 1980). Therefore, shape features could just as easily interrelate with relatively shorter duration basic effort actions, as well as longer duration transformation drives. As many more basic effort actions than transformation drives were observed in the performance material, we chose the “less than 1 s” criterion for shape feature observations shared between participants to match with that chosen for basic effort actions. Examination of the data revealed that the variability of shape feature observation start times were on average 0.4 s (SD = 0.3 s). The average duration of shape feature observations was 0.6 s (SD = 0.2 s).

According to the criterion set for the observation of shape features at the same temporal location, there were 35 locations of shared observations. All three participants made observations at five (14.29%) of these shared locations, and two participants made observations in the remaining 30 (85.71%) locations. These shared locations accounted for 75 (50.00%) of the total 150 shape features observations summed between participants. At least two participants reported observing the same type of shape feature at 27 (77.14%) of the 35 shared observational locations. Observations of the same type of shape feature at the same location accounted for 57 (76.00%) of the total 75 shape feature observations at shared locations. **Figure 6** shows the breakdown of agreement between participants at shared observational locations. The majority of disagreements as to the type of shape feature observed at shared locations (“Disagree” portions of the two shape feature columns in **Figure 6**) were due to participants reporting different aspects of movement on a single axis of space. For example, one participant would report “rising,” and another participant report “sinking” – both movements on the vertical axis of space.

## DISCUSSION

The aim of the present study was to examine the nexus between self and others’ performance evaluations. Independent analyses of a Western classical marimba player’s expressive bodily behaviors, as observed in an audio-visual recorded performance, were the focus. Self-analysis, using the effort-shape analytical system (Broughton and Stevens, 2012), was compared to analyses conducted by a Western classical percussionist, and singer – both also expert musicians. We expected that individual differences in embodied expertise would offer an explanation as to differences found between participants’ effort-shape analyses. Overall, results supported the hypothesis that observers’ effort-shape analyses differ according to marimba-playing motor expertise, and familiarity with the music performed. However, the patterns of results were different for “goal-directed” basic effort actions, “non-goal-directed” transformation drives, and shape features.

For basic effort actions, the first author (percussionist performer) and percussionist observer did not differ significantly in their frequency of observations. They also observed a greater variety of action types in a similar manner. This suggests that their marimba-playing motor expertise may have guided their perception of expressive bodily behaviors. Results could be viewed as evidence for an inverse model of perception and action (Tucker and Ellis, 1998; Trimarchi and Luzzatti, 2011; Stewart et al., 2013). It is possible that shared representations between performer and observers were facilitated by a MNS, which had been developed through sensory-motor associative learning (Heyes, 2010; Maes et al., 2014), through a substantial period of instrumental music training. However, this proposition would require systematic investigation. Though not significant, the first author reported observing more basic effort actions than the percussionist observer. The first author’s familiarity with the music, and the specific motor program required to perform the piece may have directed her attention to observe very subtle expressive bodily behaviors. Alternatively, taking a forward model perspective, the first author may have activated sensory representations from her intimate knowledge of the motor plan constructed to carry out her expressive intentions.

The singer recorded significantly fewer basic effort action observations and fewer types than the other two participants. This suggests that lacking marimba-playing motor expertise, she may have been less able to analyze “goal-directed”, or marimba-playing, expressive bodily behaviors in a way that the first author and percussionist were. From an inverse model perspective, the perception of expressive bodily behaviors directed toward the goal of playing the marimba did not activate a sensory-motor representation for the singer. However, results could also reflect a problem with the training, or implementation of the independent analysis task. Even though participants confirmed that they understood and could implement effort-shape analysis as required. During the period when the participants were conducting their analyses, a couple of difficulties in categorizing a perceived expressive bodily behavior were reported. However, all participants managed to resolve these issues to their satisfaction independently prior to submitting their final analyses.

Comparable results for agreement between participants on the same basic effort action type observation at the same temporal location were yielded using the bin and criterion setting methods. The bin method indicated that observational agreement between participants was significantly better than compared to randomly distributed observations. This suggests that participants were able to implement this facet of the effort-shape analytical system reliably. For transformation drives, only the set criterion method yielded any agreement, possibly indicating that a wider duration bin was required. However, with so few transformation drive observations made, in comparison to basic effort actions and shape features, the music material might simply not have been conducive to performing transformation drive actions. The music features allied to different effort-shape components will be explored in a subsequent study.

For shape features, the set criterion method yielded slightly better results for agreement between participants on the same type of shape feature observation at the same temporal location than the bin method. Although agreement between participants was higher for the actual, as compared to randomly distributed shape feature observations, the difference was not statistically significant. However, the disparity of observational frequencies between participants for shape features would have skewed results toward lower agreement. In this light, observing around 50% agreement between participants is not so problematic. As well as differences in participants’ observational frequencies for effort and shape categories, the patterns of agreement between the different participants for same action types possibly highlight individual differences in fastidiousness of approach to conducting the effort-shape analysis task, as well as differences in motor expertise and familiarity with the music.

Almost 90% of participants’ basic effort action observations were made at locations shared with other participants. In 70% of these cases at least two participants reported observing the same type of action at the same time. In the majority of cases in which participants reported different basic effort actions, the difference was attributable to one effort element of the three constituting the action type. This highlights that rather than being completely bipolar, the effort elements exist on a continuum and the perceiver has to make a subjective judgment as to the point of change from one end of the continuum to the other. All three participants reported the same action type in just over a third observational locations shared between the three. This might suggest that some basic effort action types were more obvious than others (e.g., “dab” and “glide”). These perhaps contained particular features, or exceeded a certain amplitude threshold (Davidson, 1993) that made them salient to an expert musician observer, in spite of being untrained in marimba playing actions. However, the first author (percussionist performer) and percussionist reported observing the same action type at the same time in the majority of cases. This suggests that they were observing and categorizing expressive bodily behaviors in a similar manner that differed from that of the singer. Therefore, beyond familiarity with the music, or music expertise, specialist instrumental training appears to have played a role in how expressive bodily behaviors were perceived.

Individual differences in fastidiousness of approach to the analysis task may have also played a role in transformation drive observations, with results showing that the singer reported none. The first author’s reporting of significantly more transformation drive observations than the percussionist observer might also just reflect the first author’s familiarity with the music. It is possible that for an observer unfamiliar with the music, and the expressive motor plan to play the piece, these “non-goal-directed” expressive bodily behaviors were not evident, or at least not very obvious.

There were a few instances of confusion in which the first author reported a transformation drive and the percussionist observer a basic effort action. These instances might have reflected the first author’s projection of her emotionally expressive intentions on to her recorded performance, thus sharpening her perception of the recorded material. Alternatively, the first author’s expressive intentions may have dominated her perception of the expressive bodily behaviors evident in the performance material. Nonetheless, these two participants’ shared observations of the same type of action at similar temporal locations in over 40% of the cases. This perhaps indicates a role for their shared motor expertise in perceiving “non-goal-directed” as well as “goal-directed”, or marimba-playing, expressive actions. However, further research is necessary to explore these ideas more fully as so few “non-goal-directed” expressive bodily behaviors were perceived in this particular performance.

While roughly half of participants’ shape feature observations were at shared locations, participants reported observing the same type of shape feature at the same location in 76% of the cases. The first author and singer were most similar in their observation and categorization of shape features. While we might have expected a higher degree of similarity between the first author and percussionist’s observations, due to their shared embodied expertise, this was not the case. Perhaps the first author’s experience with effort-shape analysis as applied here extended her perceptual capacities to recognize shaping features more readily, overcoming predispositions to attend to certain features of expressive action at the expense of others.

While once again the first author reported the most shape observations, contrary to the pattern of results for basic effort actions and transformation drives, the singer reported the second greatest number of shape features. It is possible that the singer’s vocal music expertise directed her attention to expressive postural shaping (Bellenkes et al., 1997; Doane et al., 2004; Schriver et al., 2008). It may also be the case that her attention was directed to analyze shape features at the cost of basic effort actions, and transformation drives. Likewise, the percussionist observer’s attention might have been focused on basic effort actions at the expense of noticing shape features, and transformation drives. Sensory attenuation may have played a part in the pattern of results observed. That is, overtly imitating or simulating the observed expressive bodily behaviors, as stipulated by the effort-shape analysis system, might have had an effect of attenuating perception of sensory information.

From a forward model perspective, sensory attenuation can be a consequence when the anticipated sensory effects of planned or executed action match the actual sensory inputs (Wolpert, 1997). An “efferent copy” of the motor command is made when actions are planned or executed; sensory effects of the motor command can be anticipated. Actual sensory input is then compared with the anticipated sensory effects of the motor command. If the actual and anticipated sensory inputs and effects match, sensations are canceled. A classic example of sensory attenuation is that it is very difficult to tickle yourself (Blakemore et al., 2000). Bays et al. (2005) observed that the sensation of tapping was reduced when participants self-tapped, compared to when they were tapped without moving. Using a signal-detection theory (Green and Swets, 1966) paradigm, Cardoso-Leite et al. (2010) found that a learned visuo-motor association attenuated participants’ perception of the action effect. While sensitivity to the learned visuo-motor association was reduced, sensitivity to incongruent or neutral stimuli was not. Thus, planned or executed action lead to anticipated effects of the action, which attenuate perception of actual sensory inputs.

The task in the present study was to note any moments that stood out as expressive, then analyze these using the effort-shape analytical system. This system required participants to use a combination of visual sensory information, kinesthetic feedback from imitating or simulating the observed expressive action, and introspection in their decision making processes. This process may have invoked forward models leading to sensory attenuation effects. The resulting sensory attenuation may have led participants to conclude that they had sufficiently analyzed all the expressiveness initially perceived at that moment in the performance. They may have then neglected to analyze any further associated components – shape features for the percussionist observer, and basic effort actions for the singer. A future study will systematically examine the effects of the different processes participants engage in to conduct effort-shape analysis on expressive perception.

As the participants conducted the analysis task independently and were not required to keeps notes on their approach to the task, we do not know which components of the system they analyzed first when they perceived an expressive moment. Basic effort actions may have appeared more salient to the percussionist observer, and she may have analyzed these first. Shape features may have likewise appeared more salient to the singer who may have analyzed these first. Thus, the order in which participants analyzed components of the system may have been an influential factor on the pattern of results. Furthermore, participants may have selectively attended to expressive moments from the perspective or component of the effort-shape system most salient to them. Participants’ individual instrumental or vocal music and motor expertise might have played a role in their decision-making processes about the order in which to apply the components of the effort-shape system when conducting their analyses. Therefore, a future study will address this issue by controlling for a potential order effect.

That the first author implemented all components of the effort-shape analytical system indicates that her familiarity with the music, and intimate knowledge of the expressive motor plan required to produce the performance might have affected her perception. Being most-experienced with the effort-shape system, and having trained the other study participants, the first author might have been better equipped to confidently apply the system. Alternatively, discrepancies between the first author’s analyses and those of the other two observers might reflect processing differences when observing oneself, compared to another, performing instrumental music-playing actions. This proposition could be explored experimentally in a future study. As a next step using observational methods, a further study will involve different individuals as performer and observer participants, and a separate individual to train participants in the effort-shape analytical system. In addition, the training process will also be refined in an effort to reduce, or account for individual differences in fastidiousness of approach to conducting effort-shape analysis.

It was beyond the scope of this paper to examine expressive bodily behavior in relation to features of the music, or any cognitive and physical demands on the musician to perform it. Although certain types of movement, or movement patterns might be characteristic of particular instruments (Davidson, 2012), the frequency with which they appear in performance are likely allied to features of the music score and performer’s expressive interpretation of it. Furthermore, the patterning and prevalence of expressive movement is probably also influence by the presence of a co-performer and the extra cognitive demands co-ordinating the performance with them imposes (Williamon and Davidson, 2002; Davidson, 2012). Future studies will need to work through these issues systematically to address each aspect thoroughly.

The task in the present study demanded special training, and a significant portion of a select group of participants’ time. As such, it was only possible to include three participants. Replication of the present study would of course add strength to the arguments made here. However, intensive, observational studies involving intact and ecologically valid performance stimuli, such as the one reported here, are crucial to build understanding of potentially influential factors on observers’ judgments of performance. As these factors are understood, methods can be developed to account for individual differences that affect performance evaluations (Thompson and Williamon, 2003). Using observational methods, there is relatively more scope for identifying individual differences in observer responses since the detail in the individual responses is the focus, rather than the average response across a group of participants. The cost of the observational method is the limited generalisibility of results to a population. However, this cost dissipates as further observational studies are mounted and results for a particular phenomenon are compared.

For full understanding of embodied music production and communication processes, Leman (2008) argues that a triangulated approach, which considers the perspective of the performer, observer, and, where appropriate, technology-based measures is necessary. The present study focused on the first two elements of this triangulated approach – self-perception, and others’ perception of expressive bodily behaviors in a musician’s audio-visually recorded performance. While investigation of this issue can present some methodological challenges, understanding how self-reflective performance analysis compares to others’ perception of performance is an important issue to explore. Furthermore, the investigation presented here makes a new contribution to the growing body of practice-led research in the performative research paradigm (Haseman, 2006).

In sum, self-perception of expressive bodily behaviors shared similarities and differences with those perceived by others. Results of the present study suggested that sharing instrument specific motor expertise with the performer may have enabled the observer to analyze expressive performance in a different manner to another expert observer who is not privy to similar motor expertise. This was separate to any familiarity an observer may have with the specific music performed. Furthermore, attention seemed to be directed toward different aspects of expressive bodily behaviors in accordance with different specialist instrumental or vocal training. Implications of the present study are not only scientific, but also artistic.

To understand how self and others’ perception of performance may differ is highly relevant to performance practitioners, who reflectively analyze performance aiming to improve expressive communication. Even if using seemingly reliable and valid assessment or investigative tools (Broughton and Stevens, 2012; Wrigley and Emmerson, 2013), the task of assessing or analyzing performance can be influenced by myriad individual factors. In addition to self-analysis in the practice room, musicians would be well advised to practice performing and seek critical performance feedback from a wide range of expert instrumental and vocal musicians prior to any significant assessment or performance event. The present paper provides empirical evidence for the value of this traditional pedagogical practice, which should be encouraged further and systematically supported within the performance-training context. Then, a musicians’ performance might be better placed to stimulate the broadest range of observers’ perceptual processes in a manner sublime.

## AUTHOR CONTRIBUTIONS

Mary C. Broughton was primarily responsible for the study design, data acquisition, analysis, interpretation, and drafting and revising the manuscript. Jane W. Davidson contributed intellectually to the study design, and was involved in data acquisition, interpretation, and revision of the draft manuscript.

## Conflict of Interest Statement

The authors declare that the research was conducted in the absence of any commercial or financial relationships that could be construed as a potential conflict of interest.

## Footnotes

1 http://tla.mpi.nl/tools/tla-tools/elan/

2 http://www.affective-sciences.org/webexperimentation

3 http://www.affective-sciences.org/webexperimentation
